# Synthetic Biology of Proteins: Tuning GFPs Folding and Stability with Fluoroproline

**DOI:** 10.1371/journal.pone.0001680

**Published:** 2008-02-27

**Authors:** Thomas Steiner, Petra Hess, Jae Hyun Bae, Birgit Wiltschi, Luis Moroder, Nediljko Budisa

**Affiliations:** Max Planck Institute of Biochemistry, Martinsried, Germany; University of Washington, United States of America

## Abstract

**Background:**

Proline residues affect protein folding and stability via *cis*/*trans* isomerization of peptide bonds and by the C^γ^-*exo* or -*endo* puckering of their pyrrolidine rings. Peptide bond conformation as well as puckering propensity can be manipulated by proper choice of ring substituents, e.g. C^γ^-fluorination. Synthetic chemistry has routinely exploited ring-substituted proline analogs in order to change, modulate or control folding and stability of peptides.

**Methodology/Principal Findings:**

In order to transmit this synthetic strategy to complex proteins, the ten proline residues of enhanced green fluorescent protein (EGFP) were globally replaced by (4*R*)- and (4*S*)-fluoroprolines (FPro). By this approach, we expected to affect the *cis*/*trans* peptidyl-proline bond isomerization and pyrrolidine ring puckering, which are responsible for the slow folding of this protein. Expression of both protein variants occurred at levels comparable to the parent protein, but the (4*R*)-FPro-EGFP resulted in irreversibly unfolded inclusion bodies, whereas the (4*S*)-FPro-EGFP led to a soluble fluorescent protein. Upon thermal denaturation, refolding of this variant occurs at significantly higher rates than the parent EGFP. Comparative inspection of the X-ray structures of EGFP and (4*S*)-FPro-EGFP allowed to correlate the significantly improved refolding with the C^γ^-*endo* puckering of the pyrrolidine rings, which is favored by 4*S*-fluorination, and to lesser extents with the *cis*/*trans* isomerization of the prolines.

**Conclusions/Significance:**

We discovered that the folding rates and stability of GFP are affected to a lesser extent by *cis*/*trans* isomerization of the proline bonds than by the puckering of pyrrolidine rings. In the C^γ^-*endo* conformation the fluorine atoms are positioned in the structural context of the GFP such that a network of favorable local interactions is established. From these results the combined use of synthetic amino acids along with detailed structural knowledge and existing protein engineering methods can be envisioned as a promising strategy for the design of complex tailor-made proteins and even cellular structures of superior properties compared to the native forms.

## Introduction

**Figure 1:**
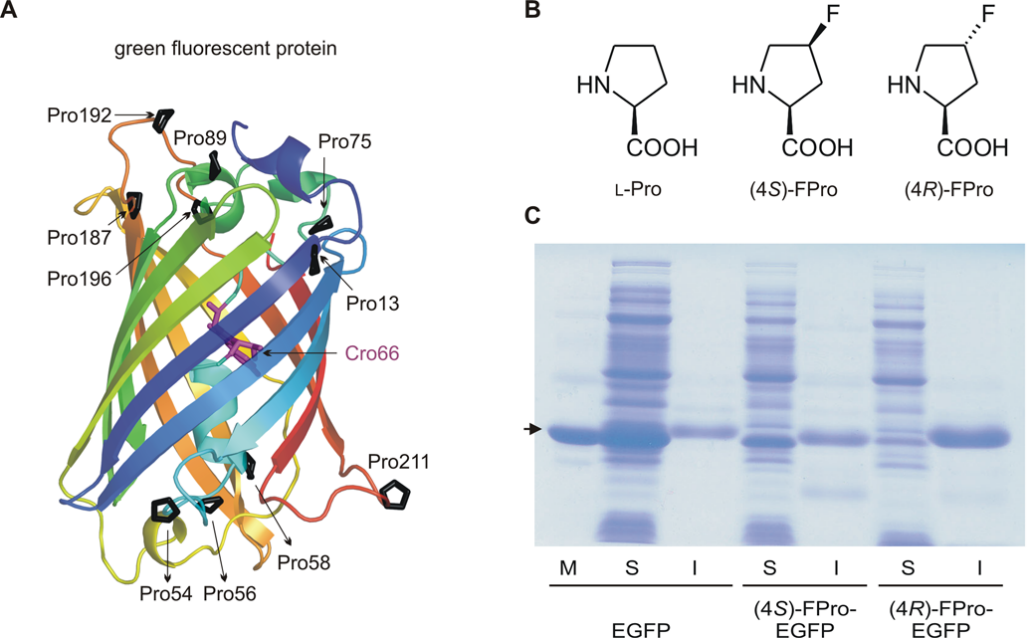
Fluoroproline variants of EGFP. (A) Characteristic β-barrel structure of EGFP with the 10 Pro residues highlighted. Cro66 indicates the fluorophore. (B) Chemical structures of proline and the two proline analogs, (2*S*,4*S*)-4-fluoroproline ((4*S*)-FPro), and (2*S*,4*R*)-4-fluoroproline ((4*R*)-FPro). (C) Expression profile of EGFP and its 4-FPro variants in *E. coli*. EGFP and (4*S*)-FPro-EGFP are predominantly soluble, whereas (4*R*)-FPro-EGFP is insoluble. Purified EGFP was applied as the molecular weight marker (M) and is indicated by the arrow; S, soluble protein fraction; I, insoluble protein fraction. Proteins were separated by SDS-PAGE and stained with Coomassie Brillant Blue.

Enhanced green fluorescent protein (EGFP) is the Phe64Leu/Ser65Thr mutant of GFP [1] (Fig. 1A) and one of the most widely used autofluorescent tags in molecular and cell biology [2]. GFPs are frequently used as reporters for both *in vitro* and *in vivo* protein folding, but their (re)folding rates are known to be very slow (10–1000 s) [2]. Therefore, an improvement of the folding properties still represents a challenge for the design and engineering of fast folding autofluorescent proteins. GFPs contain ten proline residues in their primary sequence. These prolines affect the folding rates in a decisive manner because of their known slow *cis/trans* isomerization [3]–[9]. We have therefore focused the present study on the role of these proline residues in the GFP folding process.

Among the twenty naturally occurring amino acids, proline occupies a special place. Its five-membered pyrrolidine structure causes an exceptional conformational rigidity, which is responsible for the α-helix or β-sheet disrupting properties of this residue in proteins. More importantly, *cis*/*trans* isomerization of peptidyl-proline bonds is one of the rate-determining steps in protein folding [6], [10]. The pyrrolidine ring of proline adopts two alternative conformations that differ in the position of the C^γ^ atom relative to the plane of the ring. These are referred to as either C^γ^-*exo* or C^γ^-*endo* pucker ([11], [12] and references therein). The *cis* and *trans* peptidyl-proline bond conformation and the C^γ^-*exo* and C^γ^-*endo* pucker of the pyrrolidine ring are correlated properties in proteins [11], [12], which can be affected by appropriate ring substituents such as C^γ^(C-4) fluorine atoms. Indeed, (2*S*, 4*R*)-4-fluoroproline ((4*R*)-FPro) (Fig. 1B) favors by stereoelectronic effects the *trans* conformation and C^γ^-*exo* puckering, while the epimeric (2*S*, 4*S*)-4-fluoroproline ((4*S*)-FPro) (Fig. 1B) promotes the *cis* conformation and C^γ^ *-endo* puckering [13], [14]. These properties were exploited for the synthesis of hyperstable collagen triple helices by replacing the hydroxyproline residues with (4*R*)-FPro [15], [16]. Conversely, with (4*S*)-FPro folding rates of the pseudo-wildtype barstar C40A/C82A/P27A mutant [17] were enhanced and its structure stabilized by residue-specific replacement of the single Pro48 residue with (4*S*)-FPro to favor its *cis*-conformation [13], [18]. Similarly, the folding rates of the N-terminal domain of minicollagen from *Hydra* nematocysts containing a single *cis* Pro bond were significantly and contrariwise affected by (4*R*)- or (4*S*)-FPro [19].

Based on these previous experiences it was reasonable to expect a marked effect of the two stereochemically distinct fluoroprolines (4*R*)-FPro and (4*S*)-FPro on folding and stability of EGFP where out of the 10 Pro residues 9 are involved in *trans* and only one in a *cis* peptide bond (Pro89) [20]. Upon replacement of all Pro residues in EGFP by either (4*R*)-FPro or (4*S*)-FPro we were not only able to control protein folding, but also to dissect the contributions of various factors to the folding of a complex protein molecule.

## Results and Discussion

**Figure 2:**
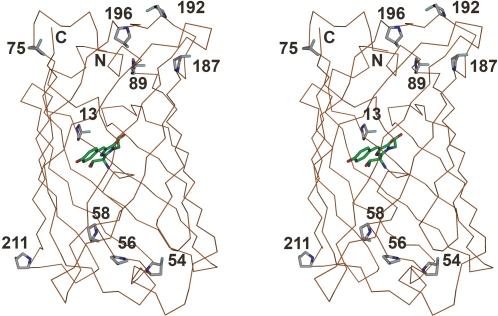
Stereo image of the crystal structure of (4*S*)-FPro-EGFP. All Pro residues were replaced by (4*S*)-FPro. Fluoroprolines (13, 54, 56, 58, 75, 89, 187, 192, 196, 211) as well as the C- and N-termini (C, N) are indicated. The chromophore is shown in green and fluorines in cyan. Note that all fluorinated Pro residues except (4*S*)-FPro56 exhibit a C^γ^-*endo* pucker. Only (4*S*)-FPro56 shows a C^γ^-*exo* puckered pyrrolidine ring.

For replacement of all 10 Pro residues in EGFP, the residue-specific method for expansion of the amino acid repertoire [21], [22] was applied. The C-terminally (His)_6_-tagged EGFP was expressed in the Pro-auxotrophic *E. coli* K-12 strain JM83, in the presence of Pro, (4*S*)-FPro and (4*R*)-FPro, respectively. The parent EGFP and the variants (4*S*)-FPro-EGFP and (4*R*)-FPro-EGFP were formed in comparably good yields (Fig. 1C). Rather surprisingly, cell pellets with (4*R*)-FPro-EGFP were colorless, indicating deposition of unfolded non-fluorescent protein in inclusion bodies. Indeed, in an SDS-gel, the (4*R*)-FPro-EGFP variant was detected exclusively in the insoluble protein fraction (Fig. 1C), and all attempts for its recovery by standard inclusion body refolding protocols failed [23]. Conversely, the parent EGFP and (4*S*)-FPro-EGFP were detected mainly in the soluble fraction as folded, fluorescent proteins (Fig. 1C). The C-terminally (His)_6_-tagged parent EGFP and (4*S*)-FPro-EGFP were purified from the soluble protein fraction and ESI-MS analysis confirmed that both proteins were isolated as monomers. In (4*S*)-FPro-EGFP all 10 Pro residues were replaced by (4*S*)-FPro (theoretical mass: 27924.5 Da; found mass: 27923.9±3.0 Da). This was further evidenced by the X-ray structure analysis of (4*S*)-FPro-EGFP (Fig. 2).

**Figure 3:**
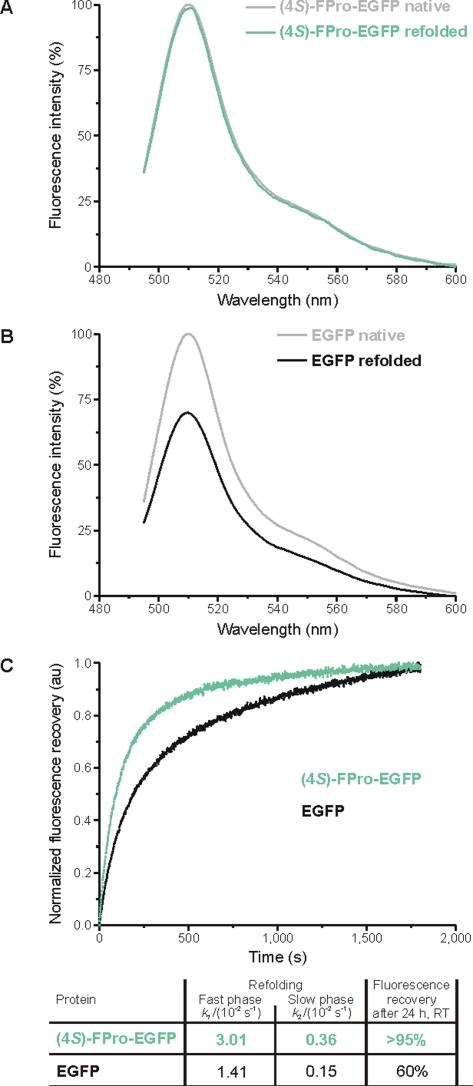
Fluorescence recovery of EGFP and (4*S*)-FPro-EGFP. The proteins were denatured by boiling (95°C, 5 min) in 8 M urea and refolded by 100-fold dilution into the buffer without urea (see Methods section for details). Fluorescence emission profiles of (A) (4*S*)-FPro-EGFP and (B) EGFP upon excitation of the chromophore at 488 nm before denaturation and after 24 h refolding at room temperature. (4*S*)-FPro-EGFP recovers more than 95% of its fluorescence before denaturation, whereas EGFP recovers only up to 60% of its initial fluorescence (this is in agreement with literature data). (C) The refolding kinetics of both proteins starts with an initial fast phase that is followed by a slow refolding phase. (4*S*)-FPro-EGFP refolds approximately 2 times faster than EGFP. The percentage of refolding was calculated on the basis of the final constant amount of fluorescence, corresponding to 100% of refolding. Normalized fluorescence in arbitrary units (au) was plotted against time.

For a comparative analysis of the folding properties of EGFP and (4*S*)-FPro-EGFP, the proteins were unfolded in boiling 8 M urea and then refolded at room temperature after 100-fold dilution into buffer. Refolding kinetics were monitored fluorometrically over a time period of at least 30 min, and the refolding efficiency was assessed after 24 h incubation under non-denaturing conditions. (4*S*)-FPro-EGFP recovered more than 95% of its fluorescence before denaturation (Fig. 3A), whereas the parent EGFP retrieved only up to 60% of its initial fluorescence (Fig. 3B). The refolding kinetics of (4*S*)-FPro-EGFP and EGFP (Fig. 3C) show an initial fast phase with rate constants of 3.01×10^−2^ s^−1^ and 1.41×10^−2^ s^−1^, respectively, that is followed by a slower refolding phase (rate constants 0.36×10^−2^ s^−1^ and 0.15×10^−2^ s^−1^, respectively; Fig. 3C). Surprisingly, (4*S*)-FPro-EGFP exhibited superior refolding properties when compared to the parent EGFP as the rate is 2.1 times faster than that of the parent EGFP in both phases. A ‘superfolder’ GFP mutant has been reported by Pédelacq *et al.* [24], which refolds upon thermal denaturation in a two-step process as well, with an initial fast rate of 5.0×10^−1^ s^−1^, which is one order of magnitude faster than that of (4*S*)-FPro-EGFP. This GFP mutant was developed from an already well-folding ‘cycle 3’ GFP mutant and relative to this parent mutant a 3.5 times enhanced folding rate was achieved.

From the results it is evident that, in contrast to our expectation, incorporation of (4*R*)-FPro into EGFP, which should favor the *trans* conformation of 9 Pro bonds, interferes with a correct protein folding, whereas (4*S*)-FPro with its opposite effect obviously does not. It is at least equally well accommodated by the protein structure as Pro and it enhances EGFP folding rates, whereas incorporation of (4*R*)-FPro apparently leads to effects that exceed the plasticity of the protein structure, and thus to irreversibly unfolded protein. A similar observation was reported recently by the group of Tirrell [25]. Indeed, global replacement of the leucine residues in GFP with 5,5,5-trifluoroleucine resulted in unfolded protein, and proper folding could only be restored after evolution of a mutant GFP that was able to accommodate the fluorinated residues [25]. In contrast to our and Pédelacq's superfolding GFP, the physical and spectroscopic properties of the fluorinated GFP mutant evolved by Tirrell's group were not superior to those of the parent GFP [25].

An inspection of the EGFP structure (PDB entry: 1EMG [20]) reveals that except Pro89 all other Pro residues are involved in *trans* peptide bonds. However, the resolution of the structure does not allow unambiguous assignment of the proline puckers. Indeed, in the process of the 3D-structure elucidation the C^γ^-*exo* or -*endo* puckering attracts little attention due to its low relevance for the overall crystallographic data quality. In folded proteins ∼5% of peptidyl-Pro bonds are in the *cis* conformation as derived by inspection of the protein structure database ([26] and references therein). In unstructured polypeptide chains the content of *cis* peptidyl-Pro bonds can reach significantly higher values particularly with aromatic amino acids directly preceding the Pro residues [9], [26], [27]. It is well known that *trans/cis* isomerization dramatically affects folding kinetics of proteins with native *cis* peptidyl-Pro bonds [5]–[9], [28]. Correspondingly, the fast initial refolding phase of EGFP should involve molecules with the Met88-Pro89 bond already in *cis* conformation whereas the slow phase should originate from denatured protein molecules with this peptide bond in *trans* [29]. In our (4*S*)-FPro-EGFP variant, the *cis* conformation of Met88-(4*S*)-FPro89 bond is favored and, thus, should accelerate the folding rates. In contrast to the experimental findings, the (4*S*)-FPro residues in all other nine positions were expected to disfavor the *trans* conformation and, thus, refolding properties. Obviously, the enhanced refolding rates of (4*S*)-FPro-EGFP have to originate from other factors.

The crystal structure of (4*S*)-FPro-EGFP (PDB entry: 2Q6P) solved at 2.1 Å resolution (Fig. 2; refinement statistics are reported in Table 1; for details on crystallization conditions see Materials and Methods) confirmed that incorporation of the 10 (4*S*)-FPro residues did not affect the overall protein fold. All (4*S*)-FPro residues display C^γ^-*endo* puckered pyrrolidine rings apart from Pro56, which adopts a C^γ^-*exo* configuration. Indeed, the fluoroprolines are well defined and characterized by low B-factors indicating rigid local conformations in the protein matrix. As outlined above, (4*S*)-fluorination of Pro promotes C^γ^-*endo* puckering. Apparently, such spatial display with preferred C^γ^-*endo* puckering of 9 out of 10 Pro residues dramatically improves the folding properties.

**Table 1:** X-ray data collection and refinement statistics.

Data collection	
Space Group	P2(1)2(1)2(1)
a (Å)	51.168
b (Å)	62.556
c (Å)	69.215
α = β = γ (°)	90
Resolution (Å)	46.6–2.1
	(2.17–2.1)
Redundancy	3.5 (2.9)
Completeness (%)	95.8 (91.5)
I/σ (I)	15 (5)
R_merge_	0.08 (0.21)
**Refinement**	
Resolution (Å)	20–2.1
No. of reflections	12901
R_crys_ a	0.227 (0.233)
R_free_ b	0.260 (0.264)
Protein atoms in the asym. Unit	1807
Solvent content (%)	43.4
Solvent atoms	117
Protein B-factor (Å^2^)	15.6
R.m.s.d. bond lengths (Å)	0.013
R.m.s.d. bond angles (°)	1.7
Ramachandran φ/ψ distribution (%)^c^	87.9/12.1/0/0
PDB entry	2Q6P

Values for the highest resolution shell are given in parentheses.

a R_crys_ = Σ|F (obs)–F (calc)|/ΣF (obs).

b R_free_ was determined from 10% of the data that were omitted from the refinement.

c Ramachandran plot distribution refers to the most favored/additional/generously/disallowed regions as defined by Procheck [38].

**Figure 4:**
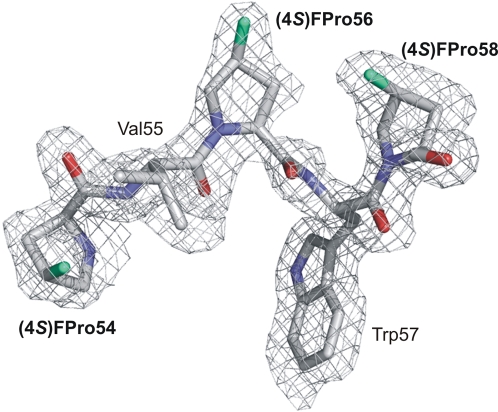
X-ray structure of the proline-rich pentapeptide (4*S*)-FPro54-Val55-(4*S*)-FPro56-Trp57-(4*S*)-FPro58 (PVPWP). The continuous electron density (grey, 2Fo-Fc; contouring levels 1 σ) indicates fluorine atoms at the 4*S*-position in three buried Pro residues (54, 56, 58). Their experimental electron densities are localized unambiguously (image preparation with PYMOL (http://pymol.sourceforge.net/)). Out of the three Pro residues forming *trans* peptide bonds, only Pro56 exhibits predominant C^γ^-*exo* pucker whereas the other two have pyrrolidine rings with C^γ^-*endo* conformation. The rigid local secondary structure of this motif forces the (4*S*)-fluorinated pyrrolidine ring of (4*S*)-FPro56 into a stereochemically unfavorable C^γ^-*exo* pucker.

Among the 10 Pro residues, five (13, 75, 89,192 and 211) are surface-exposed in EGFP, one is partially exposed (187) and the other residues are buried in the protein core (54, 56, 58 and 196). Fluorination of the buried residues increases their hydrophobicity and thus stabilizes the folded protein. We observed that the fluorinated EGFP was less prone to aggregation over the time and that related samples crystallized faster (overnight) than those of the parent protein (few days). Three of the buried proline residues are located in the characteristic proline-rich pentapeptide (4*S*)-FPro54-Val55-(4*S*)-FPro56-Trp57-(4*S*)-FPro58 (PVPWP motif; Fig. 4). The average B-factors for the prolines in the PVPWP motif as well as of the chromophore atoms are generally low (∼12 Å^2^ in (4*S*)-FPro-EGFP). Furthermore, in the crystalline state of (4*S*)-FPro-EGFP neighboring residues of fluorinated PVPWP exhibit lower average B-factors (∼5–7 Å^2^) as well.

The presence of the PVPWP pentapeptide in the GFP sequence has long been recognized [30], however, its significance is still unclear. Searching different protein databases (SwissProt, NCBI databases) we found the PVPWP motif in various proteins as different as cytochromes and eukaryotic voltage-activated potassium channels. Furthermore, we observed that the PVPWP motif is crucial for the EGFP function since site directed mutagenesis of Val55 abolishes protein fluorescence (unpublished data). Similarly, Trp57 cannot be replaced by any of the other 19 amino acids [31]. Thus, we speculate that the function of this proline-rich pentapeptide in GFP is to control the spatial orientation of the relatively bulky hydrophobic Val55 and Trp57 side chains. This is required for protecting the fluorophore from collisional quenching, e.g., by oxygen or other diffusible ligands.

**Figure 5:**
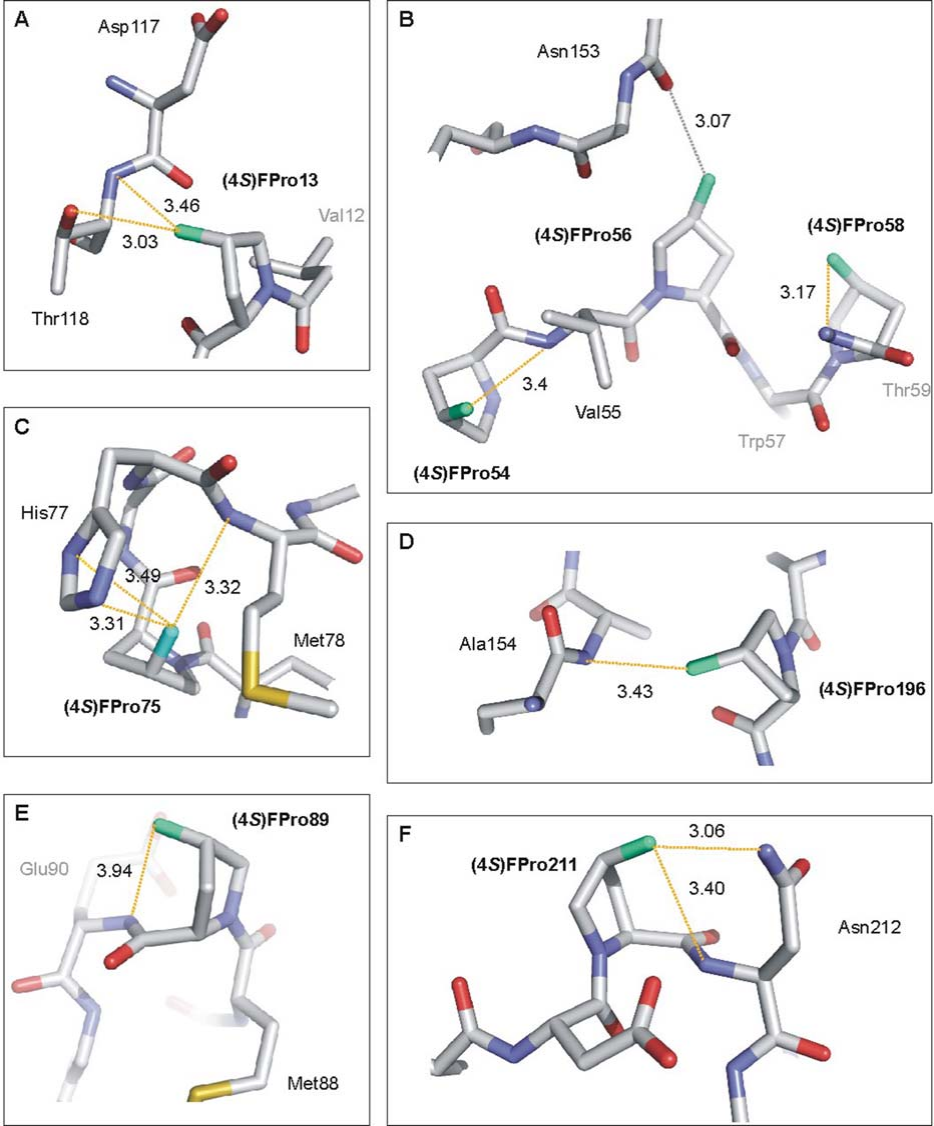
Local microenvironments of the fluorinated prolines in (4*S*)-FPro-EGFP. The high resolution (2.1 Å) X-ray crystallographic structure of (4*S*)-FPro-EGFP allowed identification of new interactions introduced by 4*S*-fluorination. The fluorine atoms are characterized by well defined electron densities at the H→F replacement sites and facilitated unambiguous determination of the conformation of the pyrrolidine rings (see also Fig. 2). Fluorines are cyan, the new interactions are shown in yellow except one repulsive interaction, which is indicated in grey. All images were prepared with PYMOL (http://pymol.sourceforge.net/). (A) (4*S*)-FPro13 interacts with the backbone -NH- of Asp117 (3.46 Å) and with Oγ2 of Thr118 (3.03 Å) on the neighboring strand. (B) The fluorinated PVPWP motif: the 4*S*-fluorine of (4*S*)-FPro56 is in a stereochemically unfavored position; it is most probably involved in a repulsive interaction with the backbone carbonyl group of Asn153 on the neighboring strand (measured crystallographic distance: 3.07 Å). The other two fluorinated prolines are involved in dipole interactions with neighboring backbone -NH- groups: (4*S*)-FPro54 with Val55 (3.40 Å) and (4*S*)-FPro58 with Thr59 (3.17 Å). (C) (4*S*)-FPro75 interacts with the backbone -NH- of Met78 (3.32 Å) and establishes a contact (∼3.4 Å) with the -NH- of the His77 imidazole ring. (D) (4*S*)-FPro196 interacts with the backbone -NH- of the adjacent Ala154 (3.43 Å), and (E) (4*S*)-FPro89 with that of the succeeding Glu90 (3.46 Å). Finally, (F) (4*S*)-FPro211 interacts with both, the backbone -NH- (3.40 Å) and Nб (3.06 Å) of the succeeding Asn212. In total, the fluorine atoms in (4*S*)-FPro-EGFP establish 12 novel interactions that are absent in EGFP.

The (4*S*) H→F replacement in Pro residues endows the pyrrolidine rings with large dipole moments because of the highly polar C–F bonds. This may promote strong dipole interactions in the local environments with polar groups such as amides, hydroxy or carbonyl groups. Indeed, 12 new interactions were detected in the (4*S*)-FPro-EGFP structure that were not present in EGFP (see Fig. 5). The majority of the fluorine atoms in (4*S*)-FPro-EGFP is involved in interactions with hydrogen atoms from neighboring backbone -NH- groups on their ‘own’ strand or on strands in the near vicinity (Fig. 5A–F). Only for the 4*S*-fluorine atoms at positions 187 and 192 direct interactions could not be detected. As outlined above, (4*S*)-FPro56 is the only fluoroproline having a C^γ^-*exo* pucker. This puckering directs the (4*S*)-fluorine atom towards an unfavorable position as it is involved in a repulsive interaction (3.07 Å) with the backbone carbonyl group of Asn153 on the neighboring strand (Fig. 5B). However, the destabilizing effect of this repulsion is apparently largely outweighed by the other stabilizing interactions. The crystallographic distances detected in the (4*S*)-FPro-EGFP structure are well compatible with C–F—H–N/O electrostatic interactions, which are more favorable in the *endo* than they would be in the *exo* pucker conformation. We are well aware that hydrogen bonding to organic fluorine is a matter of considerable controversy [32]; thus, higher resolution three-dimensional structures of fluorinated proteins are required to shed more light on this disputed matter.

It is obvious that fluorination of the Pro residues in EGFP is the main source of the superior refolding rates which may originate from several synergistic effects. The energy difference between the *cis* and *trans* Pro bond conformation is significantly larger for the *exo* than for the *endo* pucker and the activation energy for the *cis*→*trans* isomerization of (4*S*)-FPro is almost identical to that of Pro [14]. Correspondingly, the *cis*/*trans* isomerization of the proline bonds affects folding of the EGFP variant to significantly lesser extents than the preferred *endo* puckering of the (4*S*)-FPro pyrrolidine ring. In the structural context, this generates stabilizing interactions of the fluorine atoms in (4*S*)-FPro-EGFP that are absent in the parent EGFP. Conversely, the “superfolding GFP” of Pédelacq *et al.* [24] was generated by random mutagenesis of amino acids that likewise resulted in intramolecular interaction networks not present in native GFP.

Although our study reports a serendipitous discovery, we are convinced that the insights we gained here can be generally useful for the design and engineering of proteins where proline residues play decisive roles. We think that the stability of a protein can be rationally manipulated by choosing the appropriate amino acid to fit into the 3D fine structure of the target protein. Alternatively, the structure of a target protein can be optimized for the incorporation of synthetic amino acids by guided evolution [25]. Thus, detailed protein models and classical engineering methods in combination with an expanded genetic code could open a new era of synthetic biology.

## Materials and Methods

### Chemicals, analog incorporation, fermentation and protein purification

The amino acids Pro, (4*R*)-FPro and (4*S*)-FPro were purchased from Bachem AG (Bubendorf, Switzerland). Unless otherwise stated all chemicals were from Sigma (Steinheim, Germany) or Merck (Merck KGaA, Darmstadt, Germany). The Pro-auxotrophic *Escherichia coli* K-12 strain JM83 from ATCC (catalogue number 35607; genotype: F^−^ Δ(*lac*-*pro*AB) φ80 Δ(*lac*Z) M15 *ara rps*L *thi* λ^−^) served as the host organism for expression experiments. The EGFP expression plasmid was constructed as follows: The *Nco*I-*Hind*III fragment form the pEGFP plasmid (BD Biosciences, San Jose, CA) comprising the complete EGFP (Phe64Leu/Ser65Thr) coding sequence (with 10 coding triplets for the amino acid proline) was inserted into the pQE60 vector (Qiagen, Hilden, Germany) cleaved with the same enzymes. EGFP is expressed with a C-terminal (His)_6_-tag from the resulting expression vector pQE60-EGFP. Cells were routinely co-transformed with pREP4 (Qiagen) encoding the repressor gene *lacI^q^* and pQE60-EGFP (lacking *lacI^q^*). Transformed host cells *E. coli* JM83 were grown in New Minimal Medium [33], [34] (NMM) which contains 22 mM KH_2_PO_4_, 50 mM K_2_HPO_4_, 8.5 mM NaCl, 7.5 mM (NH_4_)_2_SO_4_, 1 mM MgSO_4_, 20 mM glucose, 1 µg/ml Ca^2+^, 1 µg/ml Fe^2+^, 0.001 µg/ml trace elements (Cu^2+^, Zn^2+^, Mn^2+^, MoO_4_^2−^), 10 µg/ml thiamine and 10 µg/ml biotin and the appropriate antibiotics (100 µg/ml ampicillin and 70 µg/ml kanamycin). The cells were first grown in NMM in the presence of 0.05 mM Pro as the natural substrate until its depletion from the culture in the mid-logarithmic growth phase (OD_600_ 0.5–0.8) as described elsewhere [34]. At that point, 1 mM of the non-canonical analog was added and at the same time the translation of the target gene product induced. Expression of EGFP in the presence of (4*S*)-FPro and (4*R*)-FPro produced proteins in yields similar to that of the parent EGFP protein (10–30 mg/L), although (4*R*)-FPro-EGFP was deposited in inclusion bodies. EGFP and its variant (4*S*)-FPro-EGFP were purified by two successive chromatographic steps: (i) Ni-NTA agarose (Qiagen) followed by elution with an imidazole gradient (0–100 mM) in 100 mM Na-phosphate buffer pH 8.0 and 0.5 M NaCl, and (ii) phenyl-sepharose (GE Healthcare Bio-Sciences AB, Uppsala, Sweden) eluted with an ammonium sulphate gradient 20–0% in 20 mM TrisCl, pH 8.0 and 1 mM EDTA.

### Crystallization and Structure Elucidation

The (4*S*)-FPro-EGFP was crystallized under the same conditions as the parent protein: (4*S*)-FPro-EGFP (16 mg/ml) was crystallized in 0.2 M Mg^2+^ acetate, 0.1 M sodium cacodylate, pH 6.5, and 13% (w/v) polyethylene glycol 8000 using the sitting drop vapor diffusion method. 2 µl of protein solution were mixed with 1 µl of precipitant solution at 20°C. The structure of the EGFP variant was solved by the molecular replacement technique using 1EMG [20] as a model. The data set was collected on an X-ray image plate system (Mar Research, Hamburg, Germany) using CuKα-radiation generated by a Rigaku rotating anode at 100°K. Crystals were transferred to their mother solution containing 20% (v/v) glycerol as a cryo-protectant and shock-frozen in a nitrogen stream.

Reflections were integrated with the program DENZO, scaled and reduced using SCALEPACK [35]. Model building and refinement was performed with CNS [36]. The initial model was refined by alternating automatic minimization protocols performed with CNS inspecting visual electron density map and manually adjusted using the program O [37]. Except for a small part of the N- and the C-terminus the whole model (G4-I229) could be built. The data collection and refinement statistics are presented in Table 1. Accession codes: Protein Data Bank: Coordinates and structure factor amplitudes were deposited with accession code 2Q6P.

### UV-absorbance and fluorescence of proteins

UV-absorption spectra of the proteins in phosphate buffered saline (PBS; 137 mM NaCl, 2.7 mM KCl, 4.3 mM Na_2_HPO_4_·7H_2_O, 1.4 mM KH_2_PO_4_, pH∼7.3) were recorded at 20°C on a Perkin-Elmer Lambda 17 UV/VIS spectrophotometer. Fluorescence spectra were excited at 488 nm by using excitation/emission slits of 5.0 nm and were recorded on a Perkin-Elmer spectrometer (LS50B) equipped with digital software. Protein concentrations were determined as described elsewhere [34].

### Denaturation and refolding of the different GFP variants

GFPs are generally conformationally very stable proteins once their structures have formed. Their denaturation only occurs under extremely harsh conditions, e.g., strong denaturants in combination with high temperature. Denaturation of purified (4*S*)-FPro-EGFP and EGFP (30 µM each) was performed in PBS containing 8 M urea and 5 mM DTT for 5 min at 95°C. Urea-denatured samples were renatured at room temperature by 100-fold dilution into PBS with 5 mM DTT but without urea. Protein refolding was monitored for 30 min by fluorescence recovery at 509 nm by using the option ‘Timedrive’ of Perkin-Elmer spectrometer (LS50B) with an interval of 3 sec and a slit of 2.5 nm. The concentrations of denatured proteins were adjusted so that the dilution yielded about 0.3 µM protein. Raw data were imported into Origin 6.1 (OriginLab Corporation, Northampton, MA) and normalized before plotting. Data were fitted with Sigma Plot (Systat Software Inc., San Jose, CA) using equations as described elsewhere [24].

In order to assess the end point fluorescence recovery of EGFP and (4*S*)-FPro-EGFP, fluorescence spectra were recorded before denaturation and after renaturation at room temperature for 24 h.
